# *ABCB1* rs2032582 variant is potentially linked withScutellariae Radix-induced liver injury: A case report

**DOI:** 10.1097/MD.0000000000046431

**Published:** 2025-12-19

**Authors:** Yiwen Tang, Lihong Fu, Xinrui Ren, Kun Liu, Xuehua Sun, Xin Zhang, Man Li, Qian Li, Wanchun Zhu, Yu Cui, Zhi Shang, Lingying Huang, Yueqiu Gao

**Affiliations:** a Laboratory of Cellular Immunology, Shanghai Shuguang Hospital Affiliated to Shanghai University of TCM, Shanghai, China; b Department of Hepatology, Shanghai Shuguang Hospital Affiliated to Shanghai University of TCM, Shanghai, China; c Department of Pathology, Shanghai Shuguang Hospital Affiliated to Shanghai University of TCM, Shanghai, China; d Department of Science and Technology, Shanghai Shuguang Hospital Affiliated to Shanghai University of TCM, Shanghai, China.

**Keywords:** idiosyncratic drug-induced liver injury, pharmacogenomics, Scutellariae Radix, variant

## Abstract

**Rationale:**

Idiosyncratic drug-induced liver injury is characterized by its unpredictability, lack of dose-dependence, and interindividual variability. Scutellariae Radix is a natural product widely used to treat liver diseases and has been rarely associated with liver injury in clinical settings. The report presents the case of a 50-year-old female who was hospitalized due to a Scutellariae Radix-induced liver injury.

**Patient concerns:**

The patient presented to our emergency department with a fever. And she was subsequently admitted to the hepatology department after being diagnosed with liver dysfunction.

**Diagnoses:**

Based on the exclusion of other potential causes and the patient’s prior similar episode of liver injury, a diagnosis of Scutellariae Radix-induced liver injury was established.

**Interventions:**

The patient stopped taking the herbal decoction and received hepatoprotective therapy.

**Outcomes:**

Levels of alanine transaminase and aspartate aminotransferase decreased by more than 50% on the eighth day and basically returned to normal at the time of discharge. The patient then followed up regularly, and her liver function remained within normal ranges.

**Lessons:**

The report describes a rare case of IDILI induced by Scutellariae Radix, and the presence of the rs2032582 variant in ABCB1 was revealed in this patient, which may represent an individual observation of potential relevance. Future studies are warranted to explore the potential genetic predisposition in larger cohorts.

## 1. Introduction

Drug-induced liver injury (DILI) refers to liver injury resulting from drug and/or its metabolite. It is categorized into direct, idiosyncratic and indirect hepatotoxicity types based on its mechanism. Idiosyncratic drug-induced liver injury (IDILI) is mainly related to individual predisposition such as immunologic and metabolic factors, and is unpredictable regarding pharmacological mechanisms and dose-independent. In contrast, direct hepatotoxicity exhibits clear dose-dependency and predictability. Indirect liver injury refers to DILI resulting from the alteration of original liver disease or host immunity by drugs.^[1]^ Among them, the diagnosis and prevention of IDILI present significant clinical challenges owing to its low incidence rate and distinctive characteristics.

Natural products have gained global popularity, with significantly increased use over the past 3 decades.^[2]^ Many consumers preferentially choose natural products under the impression that “natural = safe.” However, as consumption has risen, there has been growing awareness that natural products have the potential to induce liver injury, which is often idiosyncratic in nature.^[3]^ Therefore, epidemiological studies have estimated its incidence, ranging from approximately 1.16 to 6.38 per 100,000 in different regions.^[2]^ Among the numerous natural products, Scutellariae Radix has been gradually associated with IDILI.

Scutellariae Radix, known as “Huangqin” in traditional Chinese medicine, has been used for centuries to treat febrile diseases, liver disorders, and gastrointestinal inflammation. It is typically administered in decoctions or compound formulas, with daily crude herb doses ranging from 3 to 10 grams,^[4]^ although modern preparations vary considerably in concentration and duration of use. Baicalin, its major active flavonoid, has been reported to exhibit anti-inflammatory, anti-bacterial, antifibrotic, and antitumor activities in various in vitro and animal models.^[5–8]^ However, its pharmacological effects are known to be dose-dependent, and the translation of preclinical findings to human settings remains limited. Currently, accumulating clinical reports suggest that under certain conditions, excessive or prolonged use of Scutellariae Radix or its extracts may be associated with liver injury. In the 2023 AASLD Practice Guidance, Scutellariae Radix was listed for the first time as a natural product associated with hepatotoxicity.^[9]^ The inclusion of Scutellariae Radix likely reflects its repeated implication in hepatotoxicity case series and adverse event databases, despite the lack of specific references in the guideline itself.^[10–12]^ Here, we report a rare case of IDILI caused by Scutellariae Radix, marking the patient’s second occurrence of Scutellariae Radix-induced liver injury (SRILI). Given the extremely low incidence of SRILI and exclusion of potential causes, genetic testing was performed, identifying a rare genetic variant that may be implicated in SRILI pathogenesis, though further population-based studies are needed to confirm this observation.

## 2. Case presentation

In August 2023, a 50-year-old Han Chinese female presented to our emergency department with a fever. She was subsequently admitted to hepatology department after being diagnosed with liver dysfunction. Two weeks prior to admission, routine liver function tests were performed at our hospital, with the following results: alanine transaminase (ALT): 25 U/L; aspartate aminotransferase (AST): 25 U/L; γ-glutamyl transpeptidase (γ-GT): 46 U/L; alkaline phosphatase (ALP): 81 U/L; total bilirubin (TBil): 9.7 µmol/L, albumin: 42.4 g/L. The patient developed a fever approximately 3 hours after ingesting an herbal decoction one day before admission containing Scutellariae Radix 10 g, Gypsum Fibrosum 15 grams, Anemarrhenae Rhizoma 6 grams, Persicae Semen 9 grams, Coptidis Rhizoma 3 grams, Trionycis Carapax 10 grams, Bletillae Rhizoma 6 grams, Codonopsis Radix 10 grams, Paeoniae Radix Alba 10 grams, Platycodonis Radix 6 grams, Cinnamomi Ramulus 6 grams (water decoction reduced to 300 mL). The decoction had been prescribed and prepared by a licensed Traditional Chinese Medicine hospital and was taken 1 hour after breakfast. Her highest recorded temperature at home was 38.5°C, and she had not taken any antipyretic medication. Physical examination findings included a body temperature of 38.7°C. Auscultation of both lungs and heart revealed clear sounds, with a regular heart rate and rhythm. Her abdomen was soft, without tenderness or rebound tenderness. Murphy sign was negative, and the liver was not palpable. No obvious skin or scleral icterus was observed. Further laboratory and imaging examinations were performed. ALT, AST, and γ-GT were all 5 times above the upper limit of normal. Viral hepatitis, autoimmune liver diseases and other potential causes were excluded. The magnetic resonance elastography indicated liver fibrosis. Results are summarized in Tables 1 and 2. The patient had been taking Euthyrox 25 μg daily for 7 years due to Hashimoto thyroiditis with hypothyroidism. No other medications or supplements besides Euthyrox and herbal decoction were used. She exhibits good nutritional status with a normal body mass index. She denied blood transfusion or allergies, and reported no family history of inherited liver disorders or drug hypersensitivity. She also had no history of smoking, alcohol consumption or drug abuse.

**Table 1:** Laboratory examination in 2023.

Parameter	Value	Normal range
Liver function		
Total bilirubin (TBIL) in µmol/L	44.9	0–23
Direct bilirubin in µmol/L	21.3	0–4
Aspartate aminotransferase (AST) in U/L	598	13–35
Alanine transaminase (ALT) in U/L	601	7–40
Alkaline phosphatase (ALP) in U/L	133	50–135
γ-glutamyl transpeptidase (γ-GT) in U/L	368	7–45
Lactate dehydrogenase in U/L	715	120–250
Albumin in g/L	39.3	40–55
Kidney function		
Serum creatinine in µmol/L	57	41–73
eGFR in mL/(min × 1.73 m^2^)	102.94	>90
Uric acid in µmol/L	343	155–357
Serum urea in mmol/L	4.1	2.6–7.5
Autoimmune liver disease antibodies		
Anti-nuclear antibody	(−)	(−)
Anti-mitochondrial antibody	(−)	(−)
Anti-mitochondrial-M2 antibody	(−)	(−)
Anti-smooth muscle antibody	(−)	(−)
Anti-liver-kidney microsomal type 1 antibody	(−)	(−)
Anti-liver cytosol type 1 antibody	(−)	(−)
Anti-soluble liver antigen/liver pancreas antigen	(−)	(−)
Anti-GP210 antibody	(−)	(−)
Anti-Sp100 antibody	(−)	(−)
Immunoglobulin G in g/L	15	7.20–16.9
Immunoglobulin A in g/L	1.68	0.69–3.82
Immunoglobulin M in g/L	1.68	0.63–2.80
Virological indicators		
Hepatitis A virus: anti-HAV-IgM	(−)	(−)
Hepatitis B virus: HBsAg, anti-HBc-IgM, HBV-DNA	(−)	(−)
Hepatitis C virus: anti-HCV, HCV-RNA	(−)	(−)
Hepatitis D virus: HDAg	(−)	(−)
Hepatitis E virus: anti-HEV-IgM, anti-HEV-IgG, HEV-RNA	(−)	(−)
Cytomegalo virus: anti-CMV-IgM, anti-CMV-IgG	(−)	(−)
Epstein-barr virus: anti-EBV-IgM, anti-EBV-IgG	(−)	(−)
Syphilis	(−)	(−)
Human immunodeficiency virus	(−)	(−)
Coagulation function		
Prothrombin time in s	13.4	10.4–12.7
International normalized ratio	1.18	0.85–1.15
D-DIMER in µg/mL	2.28	0–0.55
Thyroid function		
Triiodothyronine in nmol/L	0.84	1.3–3.1
Free triiodothyronine in pmol/L	2.44	3.1–6.8
Thyroxine in nmol/L	70.44	66–181
Free thyroxine in pmol/L	12.9	12–22
Thyroid-stimulating hormone in nIU/mL	2.02	0.27–4.2
Routine blood		
Neutrophil in ×10^9^/L	4.83	2–7
Lymphocyte in ×10^9^/L	0.17	0.8–4
Eosinophil in ×10^9^/L	0	0.02–0.5
Basophil in ×10^9^/L	0.01	0–0.01
Monocyte in ×10^9^/L	0.13	0.12–1
Platelet in ×10^9^/L	162	101–320
Leukocyte in ×10^9^/L	5.14	3.69–9.16
Erythrocyte in ×10^12^/L	4.69	3.68–5.13
C-reactive protein in mg/L	7.67	0–8
Other		
Ceruloplasmin in mg/dL	35.2	22–58
Alpha-fetoprotein in ng/mL	2.64	0–8.78

**Table 2:** Imaging examination in 2023.

Imaging examination	Result
Magnetic resonance elastography	Liver fibrosis (elasticity value: 2.41–2.85 kPa)
Magnetic resonance imaging-proton density fat fraction	Liver fat content < 5%
Thyroid ultrasound	Mixed nodule in the right lobe of the thyroid gland - TI-RADS 3; Diffuse thyroid lesions
Cardiac ultrasonography	Normal
Electrocardiogram	Normal

Notably, the current episode reflects an inadvertent re-exposure to Scutellariae Radix. The patient experienced a similar episode of liver injury in 2021 after taking a herbal decoction containing Scutellariae Radix 6 grams, Paeoniae Radix Alba 10 grams, Atractylodis Macrocephalae Rhizoma 10 grams, Coptidis Rhizoma 3 grams, Poria 10 grams, Salviae Miltiorrhizae Radix et Rhizoma 10 grams, Ophiopogonis Radix 6 grams, Aurantii Fructus Immaturus 6 grams, Platycodonis Radix 6 grams, Cinnamomi Ramulus 3 grams, Pseudostellariae Radix 9 grams and Puerariae Lobatae Radix 10 grams (water decoction reduced to 300 mL), administered twice daily for 3 days. She developed a fever and mild abdominal discomfort, with markedly elevated liver enzymes (ALT ≥ 5 × ULN; ALP ≥ 5 × ULN). No other hepatotoxic medications were taken. Comprehensive workup excluded viral hepatitis, autoimmune liver diseases, and other potential causes. Combining expert opinions and based on a Roussel Uclaf causality assessment method (RUCAM) score of 6, this episode was classified as “probable” DILI. Details were presented in Tables 3–5. Symptoms resolved upon discontinuation of herbal products with no recurrence until the current episode.

**Table 3:** Laboratory examination after admission in 2021.

Parameter	Value	Normal range
Liver function		
Total bilirubin (TBIL) in µmol/L	25.9	0–23
Direct bilirubin in µmol/L	12.5	0–4
Aspartate aminotransferase (AST) in U/L	528	13–35
Alanine transaminase (ALT) in U/L	1089	7–40
Alkaline phosphatase (ALP) in U/L	344	35–100
γ-Glutamyl transpeptidase (γ-GT) in U/L	1166	7–45
Lactate dehydrogenase in U/L	242	120–250
Albumin in g/L	38.9	40–55
Kidney function		
Serum creatinine in µmol/L	48	41–73
eGFR in mL/(min × 1.73 m^2^)	131.73	>90
Uric acid in µmol/L	274	155–357
Serum urea in mmol/L	2.7	2.6–7.5
Autoimmune liver disease antibodies		
Anti-nuclear antibody	(−)	(−)
Anti-mitochondrial antibody	(−)	(−)
Anti-mitochondrial-M2 antibody	(−)	(−)
Anti-smooth muscle antibody	(−)	(−)
Anti-liver-kidney microsomal type 1 antibody	(−)	(−)
Anti-liver cytosol type 1 antibody	(−)	(−)
Anti-soluble liver antigen/liver pancreas antigen	(−)	(−)
Anti-GP210 antibody	(−)	(−)
Anti-Sp100 antibody	(−)	(−)
Immunoglobulin G in g/L	12.1	7.20–16.9
Immunoglobulin A in g/L	1.62	0.69–3.82
Immunoglobulin M in g/L	1.56	0.63–2.80
Virological indicators		
Hepatitis A virus: anti-HAV-IgM	(−)	(−)
Hepatitis B virus: HBsAg, anti-HBc-IgM, HBV-DNA	(−)	(−)
Hepatitis C virus: anti-HCV, HCV-RNA	(−)	(−)
Hepatitis D virus: HDAg	(−)	(−)
Hepatitis E virus: anti-HEV-IgM, anti-HEV-IgG, HEV-RNA	(−)	(−)
Cytomegalo virus: anti-CMV-IgM, anti-CMV-IgG	(−)	(−)
Epstein-barr virus: anti-EBV-IgM, anti-EBV-IgG	(−)	(−)
Syphilis	(−)	(−)
Human immunodeficiency virus	(−)	(−)
Coagulation function		
Prothrombin time in s	11.2	10.4–12.7
International normalized ratio	0.97	0.85–1.15
Thyroid function		
Thyroid-stimulating hormone in uIU/mL	1.86	0.27–4.2
Other		
Ceruloplasmin in mg/dL	41.3	22–58
Alpha-fetoprotein in ng/mL	2.88	0–8.78

Baseline values of liver function tests in 2021.

ALT: 29 U/L; AST: 30 U/L; γ-GT: 43 U/L; ALP: 97 U/L; TBil: 11.2 µmol/L.

**Table 4:** Imaging examination after admission in 2021.

Imaging examination	Result
Contrast-enhanced livermagnetic resonance imaging	Gallstone; Multiple cysts in right kidney
Thyroid ultrasound	Mixed nodule in the right lobe of the thyroid gland - TI-RADS 3; Diffuse thyroid lesions
Electrocardiogram	Normal

**Table 5:** Score of RUCAM in 2021.

Items for hepatocellular injury	Score	Result
1. Time to onset from the beginning of the drug/herb		
5–90 days (rechallenge: 1–15 days)	+2	
<5 or >90 days (rechallenge: >15 days)	+1	+1
Alternative: time to onset from cessation of the drug/herb		
≤15 days (except for slowly metabolized chemicals: >15 days)	+1	
2. Course of ALT after cessation of the drug/herb		
Percentage difference between ALT peak and N		
Decrease ≥50% within 8 days	+3	+3
Decrease ≥50% within 30 days	+2	
No information or continued drug use	0	
Decrease ≥50% after the 30th day	0	
Decrease <50% after the 30th day or recurrent increase	−2	
3. Risk factors		
Alcohol use (current drinks/d: >2 for women, >3 for men)	+1	
Alcohol use (current drinks/d: ≤2 for women, ≤3 for men)	0	0
Age ≥ 55 years	+1	
Age < 55 years	0	0
4. Concomitant drug(s)/herb(s)		
None or no information	0	
Concomitant drug/herb with incompatible time to onset	0	
Concomitant drug/herb with compatible or suggestive time to onset	−1	−1
Concomitant drug/herb known as hepatotoxin and with compatible or suggestive time to onset	−2	
Concomitant drug/herb with evidence for its role in this case (positive rechallenge or validated test)	−3	
5. Search for alternative causes	Tick if negative	Tick if not done
Group I (7 causes)		
HAV: anti-HAV-IgM	☑	□
Hepatobiliary sonography/ colour Doppler	☑	□
HCV: anti-HCV, HCV-RNA	☑	□
HEV: anti-HEV-IgM, anti-HEV-IgG, HEV-RNA	☑	□
Hepatobiliary sonography/colour Doppler sonography of liver vessels/endosonography/CT/MRC	☑	□
Alcoholism (AST/ALT ≥ 2)	☑	□
Acute recent hypotension history (particularly if underlying heart disease)	☑	□
Group II (5 causes)		
Complications of underlying disease(s) such as sepsis, metastatic malignancy, autoimmune hepatitis, chronic hepatitis B or C, primary biliary cholangitis or sclerosing cholangitis, genetic liver diseases	☑	□
Infection suggested by PCR and titer change for		
CMV (anti-CMV-IgM, anti-CMV-IgG)	☑	□
EBV (anti-EBV-IgM, anti-EBV-IgG)	☑	□
HSV (anti-HSV-IgM, anti-HSV-IgG)	☑	□
VZV (anti-VZV-IgM, anti-VZV-IgG)	☑	□
Evaluation of groups I and II		
All cause groups I and II – reasonably ruled out	+2	+2
The 7 causes of group I ruled out	+1	
6 or 5 causes of group I ruled out	0	
Less than 5 causes of group I ruled out	−2	
Alternative cause highly probable	−3	
6. Previous hepatotoxicity of the drug/herb		
Reaction labelled in the product characteristics	+2	
Reaction published but unlabelled	+1	+1
Reaction unknown	0	
7. Response to unintentional reexposure		
Doubling of ALT with the drug/herb alone, provided ALT below 5N before reexposure	+3	
Doubling of ALT with the drug(s)/herb(s) already given at the time of first reaction	+1	
Increase of ALT but less than N in the same conditions as for the first administration	−2	
Other situations	0	
Total score for the case		6

ALP = alkaline phosphatase, ALT = alanine transaminase, AST = aspartate aminotransferase, RUCAM = Roussel Uclaf causality assessment method.

**Figure 1.**
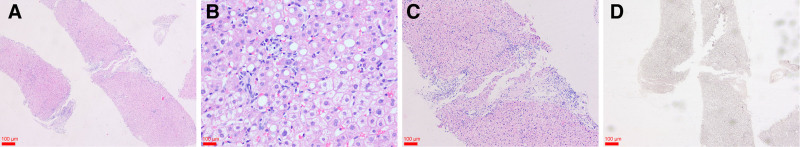
Pathological pictures of liver tissues. (A) Hepatic lobule structure was clear, with approximately 12 portal areas identified. Partial swollen hepatocytes, multiple scattered focal necrosis (accounting for approximately 10%), and mild fatty degeneration (accounting for approximately 5%) within the hepatic lobules were observed. There was mild to moderate chronic inflammatory cell infiltration in the portal area with mild interface hepatitis. Small bile ducts and small blood vessels appeared normal. Fibrous tissue hyperplasia was partially extending into the lobules [hematoxylin-eosin staining by arrow (40×)]. (B) Scattered spotty, focal necrosis and fatty degeneration [hematoxylin-eosin staining by arrow (400×)]. (C) Fibrous tissue hyperplasia in the portal area [hematoxylin-eosin staining by arrow (100×)]. (D) The reticular fiber staining showed expansion in portal areas [arrow (40×)].

Dosage, direct hepatotoxic substances, and other causes were excluded. To clarify the diagnosis, a liver biopsy was performed. Pathological results showed partial swollen hepatocytes, multiple scattered focal necrosis (accounting for approximately 10%), and mild fatty degeneration (accounting for approximately 5%) within the hepatic lobules. Percentages are calculated from systematic evaluation. CK7, CK19 and CK34 staining demonstrated that small bile ducts and vascular structures were normal. Fibrous tissue hyperplasia was observed. According to the Scheuer scoring system, the biopsy was classified as G2-3/S2. Pathological pictures are shown in Figure 1. Based upon the aforementioned findings, DILI was considered. The RUCAM scale was also applied to assess Scutellariae Radix, scoring 10, indicating a highly probable relationship between liver injury and Scutellariae Radix. Details of RUCAM are shown in Table 6.

**Table 6:** Score of RUCAM in 2023.

Items for hepatocellular injury	Score	Result
Time to onset from the beginning of the drug/herb		
5–90 d (rechallenge: 1–15 d)	+2	+2
<5 or >90 d (rechallenge: >15 d)	+1	–
Alternative: Time to onset from cessation of the drug/herb		
≤15 d (except for slowly metabolized chemicals: >15 d)	+1	–
Course of ALT after cessation of the drug/herb		
Percentage difference between ALT peak and N		
Decrease ≥ 50% within 8 d	+3	+3
Decrease ≥ 50% within 30 d	+2	–
No information or continued drug use	0	–
Decrease ≥50% after the 30th d	0	–
Decrease <0% after the 30th d or recurrent increase	−2	–
Risk factors		
Alcohol use (current drinks/d: >2 for women, >3 for men)	+1	–
Alcohol use (current drinks/d: ≤2 for women, ≤3 for men)	0	–
Age ≥ 55 yr	+1	–
Age < 55 yr	0	0
Concomitant drug(s)/herb(s)		
None or no information	0	–
Concomitant drug/herb with incompatible time to onset	0	–
Concomitant drug/herb with compatible or suggestive time to onset	−1	−1
Concomitant drug/herb known as hepatotoxin and with compatible or suggestive time to onset	−2	–
Concomitant drug/herb with evidence for its role in this case (positive rechallenge or validated test)	−3	–
Search for alternative causes	Tick if negative	Tick if not done
Group I (7 causes)		
HAV: Anti-HAV-IgM	☑	□
Hepatobiliary sonography/ color Doppler	☑	□
HCV: Anti-HCV, HCV-RNA	☑	□
HEV: Anti-HEV-IgM, anti-HEV-IgG, HEV-RNA	☑	□
Hepatobiliary sonography/color Doppler sonography of liver vessels/ endosonography/CT/MRC	☑	□
Alcoholism (AST/ALT ≥ 2)	☑	□
Acute recent hypotension history (particularly if underlying heart disease)	☑	□
Group II (5 causes)		
Complications of underlying disease(s) such as sepsis, metastatic malignancy, autoimmune hepatitis, chronic hepatitis B or C, primary biliary cholangitis or sclerosing cholangitis, genetic liver diseases	☑	□
Infection suggested by PCR and titer change for		
CMV (anti-CMV-IgM, anti-CMV-IgG)	☑	□
EBV (anti-EBV-IgM, anti-EBV-IgG)	☑	□
HSV (anti-HSV-IgM, anti-HSV-IgG)	☑	□
VZV (anti-VZV-IgM, anti-VZV-IgG)	☑	□
Evaluation of groups I and II		
All causes-groups I and II – reasonably ruled out	+2	+2
The 7 causes of group I ruled out	+1	
6 or 5 causes of group I ruled out	0	
<5 causes of group I ruled out	−2	
Alternative cause highly probable	−3	
Previous hepatotoxicity of the drug/herb		
Reaction labelled in the product characteristics	+2	
Reaction published but unlabeled	+1	+1
Reaction unknown	0	
Response to unintentional reexposure		
Doubling of ALT with the drug/herb alone, provided ALT below 5N before reexposure	+3	+3
Doubling of ALT with the drug(s)/herb(s) already given at the time of first reaction	+1	
Increase of ALT but less than N in the same conditions as for the first administration	−2	
Other situations	0	
Total score for the case		10

ALP = alkaline phosphatase, ALT = alanine transaminase, AST = aspartate aminotransferase, RUCAM = Roussel Uclaf causality assessment method.

**Figure 2.**
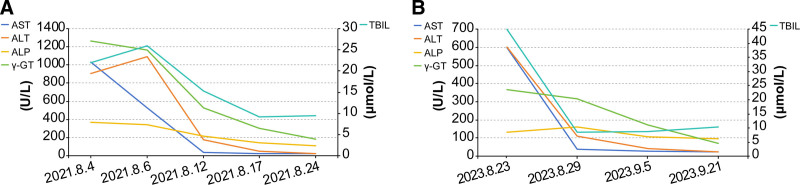
Values of liver function measured in 2021 (A) and 2023 (B). The left vertical axis represents levels of aspartate aminotransferase (AST), alanine transaminase (ALT), alkaline phosphatase (ALP) and γ-glutamyl transpeptidase (γ-GT) in units of U/L, while the right vertical axis indicates the concentration of total bilirubin (TBIL) in units of μmol/L. Horizontal axis represents different time points of measurement.

After admission, discontinuation of herbal decoction was suggested. Based on current national practice and expert consensus, the patient received intravenous glutathione (1.8 g/day), compound glycyrrhizin (160 mg/day), and polyene phosphatidylcholine (25 mL/day) for 18 days. These hepatoprotective agents are commonly used in the management of DILI in China, particularly when there is moderate to marked transaminase elevation, although randomized controlled trials remain limited.^[1,9]^ The patient demonstrated good adherence with no reported adverse events. Meanwhile, she continued to take Euthyrox 25 μg daily. Her temperature returned to normal after 3 days of treatment. ALT and AST levels decreased by more than 50% on the eighth day and basically returned to normal on her discharge (day 18). She then followed up regularly, and her liver function remained within normal ranges. After more than 3 months of clinical follow-up, the diagnosis of DILI was further confirmed. Detailed values of current and previous liver function tests are shown in Figure 2. Important milestones in 2023 are listed in Supplementary File S1 (Supplemental Digital Content, https://links.lww.com/MD/Q922).

To explore the causes, whole exome sequencing was performed. Bioinformatic analysis was conducted according to a standardized pipeline. Briefly, we performed alignment to the human reference genome using BWA-MEM, followed by variant calling with the GATK HaplotypeCaller. Stringent quality filters were applied (depth > 20×, base quality > 30, mapping quality > 40). Variants were annotated using ANNOVAR with population frequency references from 1000 Genomes Project (GRCh37) and Exome Aggregation Consortium (ExAC r0.3), as well as functional predictions from SIFT and PolyPhen-2. A total of 76,724 gene variants were identified. Of these, 56,251 were located in exons, including 28,459 non-synonymous single nucleotide variants. We then filtered for rare variants (minor allele frequency < 5%) both in *GRCh37*_all and *ExAC*_all. When frequencies differed between databases, the higher of 2 values were used. Refinement of candidate variant was based on rarity, predicted functional impact on drug metabolism/transport genes and consistency with the patient’s clinical presentation. After in-depth analysis, the top 3 genes harboring candidate variants likely associated with DILI in this case were adenosine triphosphate-binding cassette (ABC), cytochrome P450 (CYP450) and human leukocyte antigen (HLA).

## 3. Discussion

Based on clinical presentation and laboratory findings, the diagnosis of DILI was established, exhibiting a hepatocellular pattern. In this instance, Scutellariae Radix was considered the causative agent. Scutellariae Radix is a commonly used natural product worldwide. Studies have confirmed its therapeutic effects on liver and gallbladder diseases. Consequently, this case is unusual, as many patients consume herbal decoctions containing similar ingredients. It is of significant importance to investigate the underlying mechanisms and to explore strategies for prevention.

The reasons why Scutellariae Radix is the most likely causative factor are as follows. We compared 2 prescriptions. The components of these prescriptions, including both their Chinese herbal names and corresponding Latin nomenclature, are detailed in Supplementary File S2 (Supplemental Digital Content, https://links.lww.com/MD/Q922). Five identical herbs were identified, including Scutellariae Radix, Coptidis Rhizoma, Cinnamomi Ramulus, Platycodonis Radix and Paeoniae Radix Alba. Based on the preliminary research in our team, Scutellariae Radix ranked tenth in the category of single herb-induced liver injury. 48 case reports of SRILI were documented from multiple clinical centers.^[13]^ A similar case was reported in Japan: a patient experienced DILI twice after taking Scutellariae Radix,^[14]^ highlighting a potentially reproducible clinical pattern. Furthermore, Scutellariae Radix has been included in the category of natural products implicated in hepatotoxicity in 2023 AASLD Practice Guidance.^[9]^ We also retrieved information regarding hepatotoxicity of the aforementioned herbs. According to LiverTox, Scutellaria Radix is assigned a Category B likelihood score, suggesting it is a possible, but rare, cause of clinically apparent liver injury.^[15]^ However, this classification is derived from expert consensus based on a small number of case reports and lacks robust epidemiological validation. Therefore, the LiverTox rating should be viewed as hypothesis-generating. In our case, the observed consistency with previous reports may provide preliminary signal value but cannot independently establish causation. It is also noteworthy that the rechallenge of Scutellariae Radix is positive with a RUCAM score of 10 (highly possible).

SRILI is idiosyncratic, a relatively rare drug reaction. The majority of individuals taking Scutellariae Radix at the recommended dose will not result in liver damage. In the present study, susceptivity to DILI caused by herbs is associated with host factors, including genetic traits. It is well known that specific genes are strongly associated with DILI when triggered by certain medications. *ABCB*,^[16]^ *HLA*,^[17]^ *CYP450*,^[18]^ and other drug metabolism-related genes have been studied in relation to DILI. Among the candidate variants identified, rs2032582 in *ABCB1*, previously reported to affect drug metabolism and transport, may warrant further investigation given its potential pharmacogenomic relevance.

*ABCB1* is one of the *ABC* genes, also known as multi-drug resistant gene-1. The P-glycoprotein, encoded by *ABCB1*, is an important transporter and a key player in drug handling, protecting the body from toxins and exogenous substances.^[19,20]^ Rs2032582 in *ABCB1* is a non-synonymous variant in the exonic part of chromosome 7. The reference allele for rs2032582 is A (adenine), and the alternative allele is T (thymine). Rs2032582 variant in *ABCB1* results in a non-synonymous substitution from serine to threonine, which may affect P-glycoprotein structure or function. While this change has been associated with altered drug transport and susceptibility to DILI in some contexts, no direct evidence currently links this variant to hepatotoxicity from Scutellariae Radix. Therefore, we propose this as a hypothesis that warrants further investigation in functional and pharmacokinetic studies. Researches have found the relationship between rs2032582 and specific DILIs. A study in Japan shows that patients with rs2032582 have a higher risk of atorvastatin-induced liver injury.^[21]^ Similar finding has been observed in China.^[22]^ Although rs2032582 in the *ABCB1* gene has been associated with susceptibility to atorvastatin-induced liver injury via P-glycoprotein modulation, its relevance to herbal hepatotoxicity remains speculative. The metabolism of Scutellariae Radix and its major constituents (e.g., baicalin, baicalein) is not fully elucidated, and there is currently no direct evidence that these compounds undergo biotransformation via CYP450 enzymes or affect P-glycoprotein function. While the recurrence of liver injury in this patient carrying rs2032582 is notable, it should be interpreted as a potential signal requiring further pharmacogenomic investigation, rather than conclusive evidence of causality.

Rs45476795, a gene variant in *ABCB4*, was identified in this patient as well. Variants in *ABCB4* may result in ABCB4 deficiency, which predisposes to cholestatic DILI and other diseases related to impaired biliary excretion and cholestasis.^[16]^ In this case, the patient mainly exhibits hepatocellular liver injury, which is inconsistent with the type linked to *ABCB4*. Genetic polymorphisms in CYP450 enzymes have been widely implicated in DILI, particularly involving drugs metabolized by CYP2E1, CYP2C9, CYP2C19, and CYP3A4. Numerous studies have shown that variants in these enzymes can alter drug metabolism and increase susceptibility to hepatotoxicity. In the data, variants like rs528725654, rs28371759, rs201045618 and rs55901263 in *CYP450* were identified. However, no potential association has been established between these variants and SRILI. For natural products like Scutellariae Radix, the metabolic pathways and their interaction with CYP450 enzymes are not well-characterized. Therefore, while *CYP450* variants remain plausible contributors, the current case did not reveal compelling associations beyond the rs2032582 variant in *ABCB1*, which warrants further investigation in larger cohorts. For *HLA*-related variants, despite a limited number of studies reporting associations with DILI, the variants identified in these studies differ from those observed such as rs1059582, rs3180380 and rs1064944 in this patient. No clear association with DILI was observed for the other, even rarer variants not included in this analysis, as they have not been investigated in depth in previous studies. Consequently, we propose that rs2032582 in *ABCB1* may be a potential susceptibility factor in this patient, although further validation in larger populations is required.

## 4. Patient perspective

The patient expressed concern and confusion upon the recurrence of liver injury after taking herbal medicines. She reported feeling anxious but grateful for the timely diagnosis and supportive care. She acknowledged the importance of sharing her experience to raise awareness about potential risks associated with traditional herbal formulations.

## 5. Conclusions

The report describes a rare case of IDILI induced by Scutellariae Radix, and the presence of rs2032582 variant in *ABCB1* was revealed in this patient, which may represent an individual observation of potential relevance. Future studies are warranted to explore the potential genetic predisposition in larger cohorts; however, no formal validation study is currently underway at the time of this report.

## Author contributions

**Conceptualization:** Xuehua Sun, Yueqiu Gao.

**Data curation:** Yiwen Tang, Wanchun Zhu, Yu Cui.

**Formal analysis:** Kun Liu.

**Funding acquisition:** Yueqiu Gao, Xuehua Sun.

**Investigation:** Lihong Fu, Kun Liu.

**Project administration:** Xuehua Sun.

**Resources:** Lingying Huang.

**Supervision:** Xin Zhang, Man Li.

**Validation:** Xinrui Ren, Qian Li.

**Visualization:** Xinrui Ren.

**Writing – original draft:** Yiwen Tang.

**Writing – review & editing:** Lihong Fu, Zhi Shang, Lingying Huang.

## Supplementary Material


