# Supplementary material for: ABCB1 rs2032582 variant is potentially linked withScutellariae Radix-induced liver injury: A case report
Source: Medicine (Baltimore). 2025 Dec 19;104(51):e46431. doi: 10.1097/MD.0000000000046431 (PMC12727338; doi:10.1097/MD.0000000000046431)
Supplement: Supplementary file 1 [file medi-104-e46431-s001.docx]

**Supplemental Digital Content 1**

| 2023.8.22 | The patient took an herbal decoction, following which she developed a fever. |
| --- | --- |
| 2023.8.23 | The patient went to our hospital and was admitted to hepatology department due to liver dysfunction. Herbal decoction was discontinued and hepatoprotective treatments were given. |
| 2023.8.28 | Combining laboratory tests and imaging examinations, viral hepatitis, autoimmune liver diseases and other potential causes were excluded. |
| 2023.8.29 | A liver biopsy was performed. |
| 2023.9.4 | The diagnosis of SRILI was confirmed based on RUCAM score and pathological results. |
| 2023.9.5 | Laboratory results show a significant improvement in liver function. |
| 2023.9.9 | The patient was discharged from the hospital. |

**Important Milestones**

**Supplemental Digital Content 2**

**Chinese and Latin Nomenclature of Herbs Prescribed in 2023**

| **Chinese name** | **Latin nomenclature** |
| --- | --- |
| Huangqin | SCUTELLARIAE RADIX |
| Baishao | PAEONIAE RADIX ALBA |
| Huanglian | COPTIDIS RHIZOMA |
| Jiegeng | PLATYCODONIS RADIX |
| Guizhi | CINNAMOMI RAMULUS |
| Shigao | GYPSUM FIBROSUM |
| Zhimu | ANEMARRHENAE RHIZOMA |
| Taoren | PERSICAE SEMEN |
| Biejia | TRIONYCIS CARAPAX |
| Baiji | BLETILLAE RHIZOMA |
| Dangshen | CODONOPSIS RADIX |

**Chinese and Latin Nomenclature of Herbs Prescribed in 2021**

| **Chinese name** | **Latin nomenclature** |
| --- | --- |
| Huangqin | SCUTELLARIAE RADIX |
| Baishao | PAEONIAE RADIX ALBA |
| Baizhu | ATRACTYLODIS MACROCEPHALAE RHIZOMA |
| Huanglian | COPTIDIS RHIZOMA |
| Fuling | PORIA |
| Danshen | SALVIAE MILTIORRHIZAE RADIX ET RHIZOMA |
| Maidong | OPHIOPOGONIS RADIX |
| Zhishi | AURANTII FRUCTUS IMMATURUS |
| Jiegeng | PLATYCODONIS RADIX |
| Guizhi | CINNAMOMI RAMULUS |
| Taizishen | PSEUDOSTELLARIAE RADIX |
| Gegen | PUERARIAE LOBATAE RADIX |
